# Establishing a radiomics model using contrast-enhanced ultrasound for preoperative prediction of neoplastic gallbladder polyps exceeding 10 mm

**DOI:** 10.1186/s40001-025-02292-1

**Published:** 2025-02-03

**Authors:** Dong Jiang, Yi Qian, Yijun Gu, Ru Wang, Hua Yu, Zhenmeng Wang, Hui Dong, Dongyu Chen, Yan Chen, Haozheng Jiang, Yiran Li

**Affiliations:** 1 Department of Ultrasound, Eastern Hepatobiliary Surgery Hospital, The Third Affiliated Hospital of Naval Medical University, Shanghai, China; 2 Department of Pathology, Shanghai Eastern Hepatobiliary Surgery Hospital, Third Affiliated Hospital of Naval Medical University, Shanghai, China; 3 College of art and science department: medical anthropology, psychology, public health, Case Western Reserve University, Cleveland, United States; 4 Department of Anesthesiology, Eastern Hepatobiliary Surgery Hospital, The Third Affiliated Hospital of Naval Medical University, Shanghai, China

**Keywords:** Gallbladder polyps, Radiomics, Contrast-enhanced ultrasound

## Abstract

**Background:**

A key challenge in the medical field is managing gallbladder polyps (GBP) > 10 mm, especially when their nature is uncertain. GBP with a diameter exceeding 10 mm are associated with an increased risk of gallbladder cancer, making the key to their management the differentiation between benign and malignant types. The current practice, due to the inability to predict accurately, leads to excessive surgeries and ineffective follow-ups, increasing patient risks and medical burdens.

**Purpose:**

This study aims to establish an imaging radiomics model using clinical data and contrast-enhanced ultrasound (CEUS) to predict neoplastic GBP exceeding 10 mm in diameter preoperatively.

**Materials and methods:**

Data from 119 patients with GBP > 10 mm of unknown origin were analyzed. A total of 1197 features were extracted from the GBP area using conventional ultrasound (US) and CEUS. Significant features were identified using the Mann–Whitney U test and further refined with a least absolute shrinkage and selection operator (LASSO) regression model to construct radiomic features. By integrating clinical characteristics, a radiomics nomogram was developed. The diagnostic efficacy of the preoperative logistic regression (LR) model was validated using receiver operating characteristic (ROC) curves, calibration plots, and the Hosmer–Lemeshow test. CEUS is an examination based on conventional ultrasound, and conventional two-dimensional ultrasound still poses significant challenges in differential diagnosis. CEUS has a high accuracy rate in diagnosing the benign or malignant nature of gallbladder space-occupying lesions, which can significantly reduce the preoperative waiting time for related examinations and provide more reliable diagnostic information for clinical practice.

**Results:**

Feature selection via Lasso led to a final LR model incorporating high-density lipoprotein, smoking status, basal width, and Rad_Signature. This model, derived from machine learning frameworks including Support Vector Machine (SVM), Logistic Regression (LR), Multilayer Perceptron (MLP), k-Nearest Neighbors (KNN), and eXtreme Gradient Boosting (XGBoost) with fivefold cross-validation, showed AUCs of 0.95 (95% CI: 0.90–0.99) and 0.87 (95% CI: 0.72–1.0) in internal validation. The model exhibited excellent calibration, confirmed by calibration graphs and the Hosmer–Lemeshow test (P = 0.551 and 0.544).

**Conclusion:**

The LR model accurately predicts neoplastic GBP > 10 mm preoperatively. Radiomics with CEUS is a powerful tool for analysis of GBP > 10 mm. The model not only improves diagnostic accuracy and reduces healthcare costs but also optimizes patient management through personalized treatment plans, enhancing clinical outcomes and ensuring resources are more precisely allocated to patients who need surgery.

**Supplementary Information:**

The online version contains supplementary material available at 10.1186/s40001-025-02292-1.

## Key results

- The final radiomic features, post-Lasso selection, were fed into various machine-learning algorithms including SVM, LR, MLP, KNN, and XGBoost, facilitating the formulation of risk models. The subsequent development of a radiomics nomogram, which amalgamates Rad Signature with clinical indicators, exhibited an AUC of 0.95 (95% CI: 0.90–0.99). Furthermore, sensitivity, specificity, positive predictive value, negative predictive value, and overall diagnostic accuracy were established to be 94.4%, 84.6%, 63%, 98.2%, and 63%, respectively.
- CEUS radiomic signatures had an AUC of 0.89 (95% CI: 0.82–0.97), outperforming US radiomic signatures (AUC: 0.85, 95% CI: 0.75–0.94) in predicting neoplastic GBP > 10 mm, offering valuable clinical insights.

**Supplementary Information:**

The online version contains supplementary material available at 10.1186/s40001-025-02292-1.

## Background

Gallbladder polyps (GBP) encompass polypoid protrusions originating from the gallbladder’s mucosal surface. Varied incidence rates of GBP, ranging from approximately 2–5%, have been documented in different research endeavors and epidemiological investigations [1]. Influencing factors, like age, gender, gallstones, and cholecystitis, play a pivotal role in determining these rates. GBP is a lesion often found incidentally during ultrasonography, and patients may or may not exhibit symptoms of gallbladder disease. While the majority of GBP cases are benign and classified as non-neoplastic GBP, a minority might potentially metamorphose into malignant neoplastic GBP. The challenge lies in detecting malignant polyps early and performing cholecystectomy before the cancer becomes invasive, or ensuring adequate surgical excision if invasive, while avoiding unnecessary surgery in asymptomatic patients with benign conditions. Features suggesting malignancy include a polyp size greater than 10 mm, solitary lesions, growth over time, adenomatous polyps, sessile lesions, and associated gallbladder wall thickening. The risk of malignancy is higher in patients of Indian ethnicity, those older than 50 years, and individuals with primary sclerosing cholangitis or gallstone disease. Consequently, precise evaluation and decisive management are pivotal once GBP is identified [2, 3]. Small, asymptomatic polyps, especially those with a size less than 1 cm, generally necessitate systematic follow-up to keep tabs on any evolution and variations. Because even small gallbladder polyps carry a risk of malignancy [4]. For polyps exceeding 1 cm, those that cause symptoms, and those carrying an elevated risk of malignancy, surgical intervention typically emerges as a viable treatment strategy [5]. Ultrasound is capable of sensitively detecting polypoid lesions within the gallbladder, including those of minute dimensions, which underscores the necessity of ultrasound follow-up [6, 7]. However, the reduced specificity of ultrasound affects its ability to distinguish pseudopolyps from true polyps [8, 9]. This limitation highlights the importance of supplementary advanced diagnostic tools to enhance ultrasound imaging. Pre-operative tissue diagnosis is challenging, but tools such as ultrasonography, contrast-enhanced CT, endoscopic ultrasound, and regular follow-ups can help surgeons make informed decisions [10]. Furthermore, compared to the high prevalence of GBP, the incidence of gallbladder tumors is significantly lower, further emphasizing the need for accurate diagnostic methods to identify polyps that pose a genuine risk of malignancy and warrant intervention.

Contemporary research has unveiled several aspects intimately connected with neoplastic GBP. Polyps of considerable size, notably those exceeding a 1-cm diameter, have been broadly correlated with an elevated probability of transformation into gallbladder cancer, indicating a noteworthy malignant potential [11]. An American surgical study in which Polyps larger than 2 cm should be removed due to the increased risk of high-grade dysplasia and cancer [12]. An augmented incidence of neoplastic GBP conspicuously surfaces with advancing age, rendering elderly populations more susceptible than their younger counterparts. A gender discrepancy is apparent, with females being more predisposed to neoplastic GBP relative to males. Moreover, a heightened incidence of neoplastic GBP pervades individuals afflicted with gallstones, as the persistent irritation and ensuing inflammation of the gallbladder mucosa resulting from gallstones escalates the risk associated with tumorigenesis. The relationship between chronic cholecystitis, gallbladder infection, and the manifestation of neoplastic GBP has been acknowledged [13]. GBP with a diameter exceeding 10 mm is associated with an increased risk of gallbladder cancer, and the key to their management lies in distinguishing between benign and malignant types. Current practices, due to the inability to accurately predict malignancy, often lead to unnecessary surgeries and ineffective follow-ups, thereby increasing patient risks and medical burdens. In the realm of preoperative assessments, GBP presents formidable challenges owing to the inaccessibility of preoperative tissue for evaluation [8, 14]. Ultrasonography and CT scans emerge as instrumental in helping physicians delineate judicious treatment strategies. Although radionics rooted in ultrasound or CT imaging have proffered valuable predictive insights [15], no predictive framework pivoting on gallbladder CEUS has been reported in extant literature [16]. This study aims to optimize the preoperative diagnosis of GBP and reduce unnecessary medical interventions by constructing a predictive model based on radiomics and contrast-enhanced ultrasound.

Radiomics integrates medical imaging data with computational technology, enabling an enhanced comprehension of disease mechanics and facilitating the progression of personalized medicine by methodically quantifying and interpreting distinct image attributes [17]. This technique, characterized by its non-invasive nature, capability of producing multidimensional data, operational efficiency, and consistent repeatability, scrupulously extracts pertinent information from medical imaging data. Such an approach substantively contributes to formulating strategies for disease prevention, facilitating precise diagnostics, crafting judicious treatment protocols, and implementing astute evaluation processes. Moreover, it has proven to be widely applicable in conducting a nuanced differential diagnosis of GBP [18]. Deep learning, a specialized form of machine learning, endeavors to mimic the operational mechanics of human brain neural networks, focusing on deciphering and extracting intricate patterns and features embedded in expansive data sets. This method has found substantial application in the realm of clinical medical predictive modeling in recent years, thereby enhancing the reliability and accuracy of these models [19].

Consequently, the proposition centers on formulating an innovative preoperative predictive model for neoplastic GBP exceeding 10 mm, harmonizing an array of clinical attributes, US characteristics, and CEUS features, and leveraging the profound capabilities of deep learning. The intended model seeks to navigate the preliminary treatment strategies for GBP, while also anticipating subsequent external validation through the incorporation of supplemental data.

## Materials and methods

### Patient enrollment

**Fig. 1:**
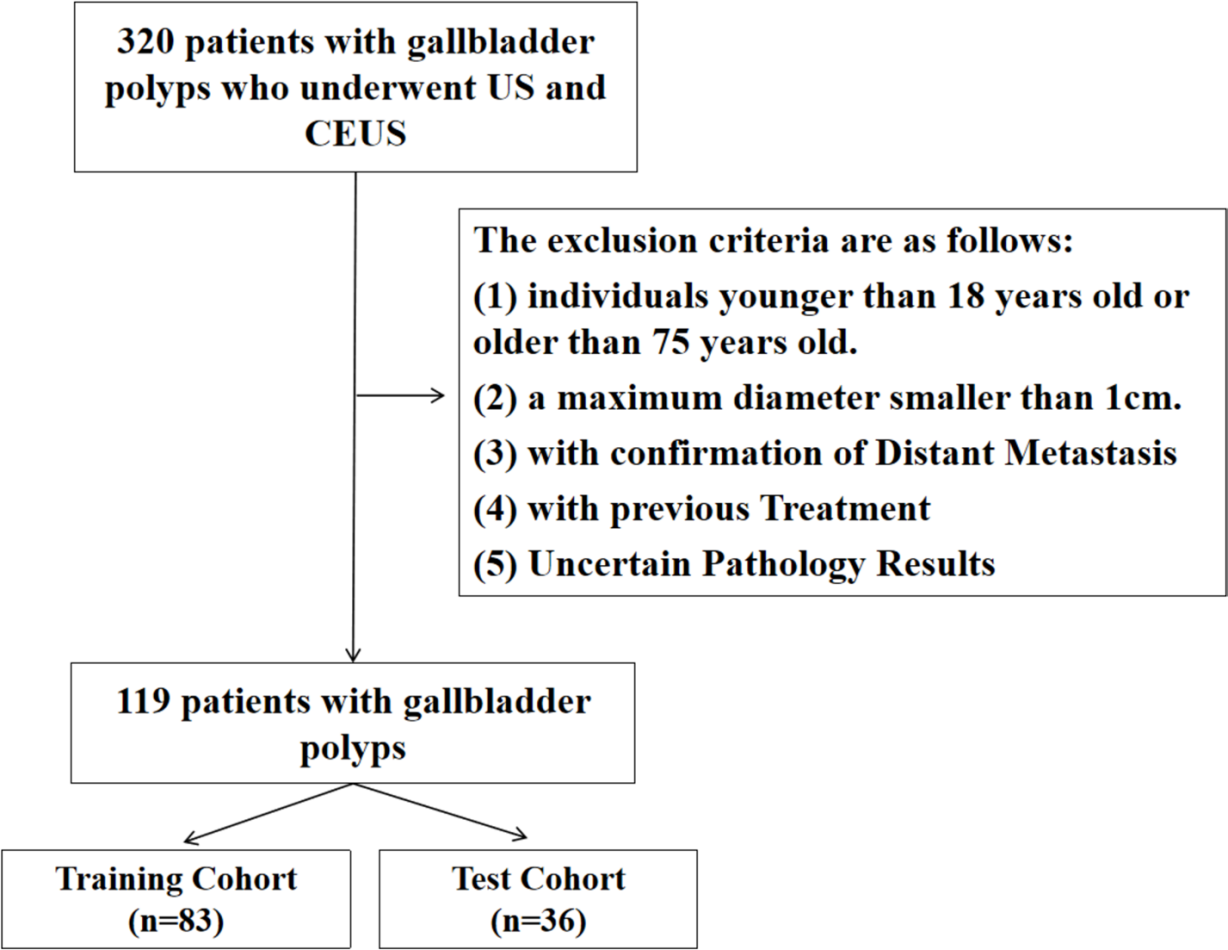
The flowchart depicted herein presents the patient enrollment process in the study

Within the timeframe spanning September 2021 to September 2023, the Eastern Hepatobiliary Surgery Hospital admitted 320 patients with GBP, all of whom underwent pathological confirmation. The establishment of exclusion criteria necessitated the omission of certain participants, specifically those below 18 or exceeding 75 years of age. The exclusion of patients over 75 years old was due to the fact that elderly individuals often experience a decline in physical function and tolerance, making them unable to withstand surgery. As a result, obtaining the pathological gold standard for a definitive diagnosis becomes challenging in this age group; those presenting with a maximum GBP diameter shy of 10 mm; individuals for whom CT or MRI substantiated the existence of distant metastasis; and those with a historical backdrop of therapeutic interventions like radiation or chemotherapy, or unclear pathological outcomes. This meticulous selection process culminated in the inclusion of 119 patients in the study, facilitating the formulation of a predictive model, and subsequent internal validation was executed utilizing a subset of 36 patients (Fig. 1). The Ethics Committee of the aforementioned hospital bestowed approval upon this retrospective study, corroborated by written informed consent acquired from all patient participants (Ethics Number: EHBHKY2023-K039-P001).

### US and CEUS examination

In our study, US served as the primary diagnostic method for assessing the size, morphology, and attachment to the gallbladder wall of gallbladder polyps, while CEUS was employed to further evaluate hemodynamic characteristics such as basal width, vascular patterns, enhancement degree, and enhancement mode. CEUS overcomes the limitations of US in detecting microvascular flow and dynamic blood perfusion, significantly improving the diagnostic accuracy in distinguishing between benign and malignant polyps. Ultrasound examinations were systematically conducted utilizing the Acuson Sequoia diagnostic ultrasound apparatus (Siemens Medical Solutions USA, Mountain View, California), employing a transabdominal 5C1 probe. Patient preparation involved a minimum fasting period of 8 h preceding the examination. The utilization of two-dimensional scans ensured accurate measurements of the length and width of each lesion, with keen observations made regarding ultrasound characteristics, including count, dimensions, morphology, demarcations, and wall thickness of GBP.

The employment of CDFI (Color Doppler Flow Imaging) facilitated the exploration of blood flow dynamics, static imagery, pedicle internal blood flow signals, and blood flow frequency spectra. Preliminary diagnoses for each lesion were determined by the ultrasound physician. Following standard ultrasound procedures, a cholecystography was initiated, involving the injection of 1.2 ml of SonoVue suspension through the antecubital vein. Optimal visualization of the polyp’s largest cross-sectional view was sought, with the dynamic imaging being preserved for 10 s at intervals of 60 s, 90 s, and 120 s to scrutinize aspects like basal width, vascular pathway, enhancement degree, and enhancement approach [20–22]. Distinct features of each GBP via abdominal US, CDFI, and CEUS comprised: maximal diameter; GBP count; presence of gallstones; US and CEUS measured stalk width of the gallbladder polyp; and existence of internal vascularity within the gallbladder polyp. For this research, the GBP stalk was characterized as the GBP segment adjoined to the gallbladder mucosal surface. Abdominal US, CDFI, and CEUS images underwent independent interpretation by a pair of physicians, one of whom possessed 5 years of abdominal CEUS expertise, and the other, over a decade of experience in abdominal CEUS. Both interpreting physicians, shielded from the patient’s clinical and pathological data, sought a collective consensus should diagnostic disparities transpire.

Although arterial-phase images (10–45 s) provide valuable insights in some diagnostic scenarios [38], our study did not specifically analyze this phase due to its rapid dynamic changes, which can make consistent imaging acquisition challenging. Moreover, our diagnostic focus was on late-phase enhancement features, such as basal width and enhancement uniformity, which have demonstrated higher specificity in differentiating neoplastic from non-neoplastic polyps. The real-time nature of CEUS already captures comprehensive enhancement dynamics, ensuring robust diagnostic evaluation.

### Segmentation of ROIs and radiomic feature extraction

**Fig. 2:**
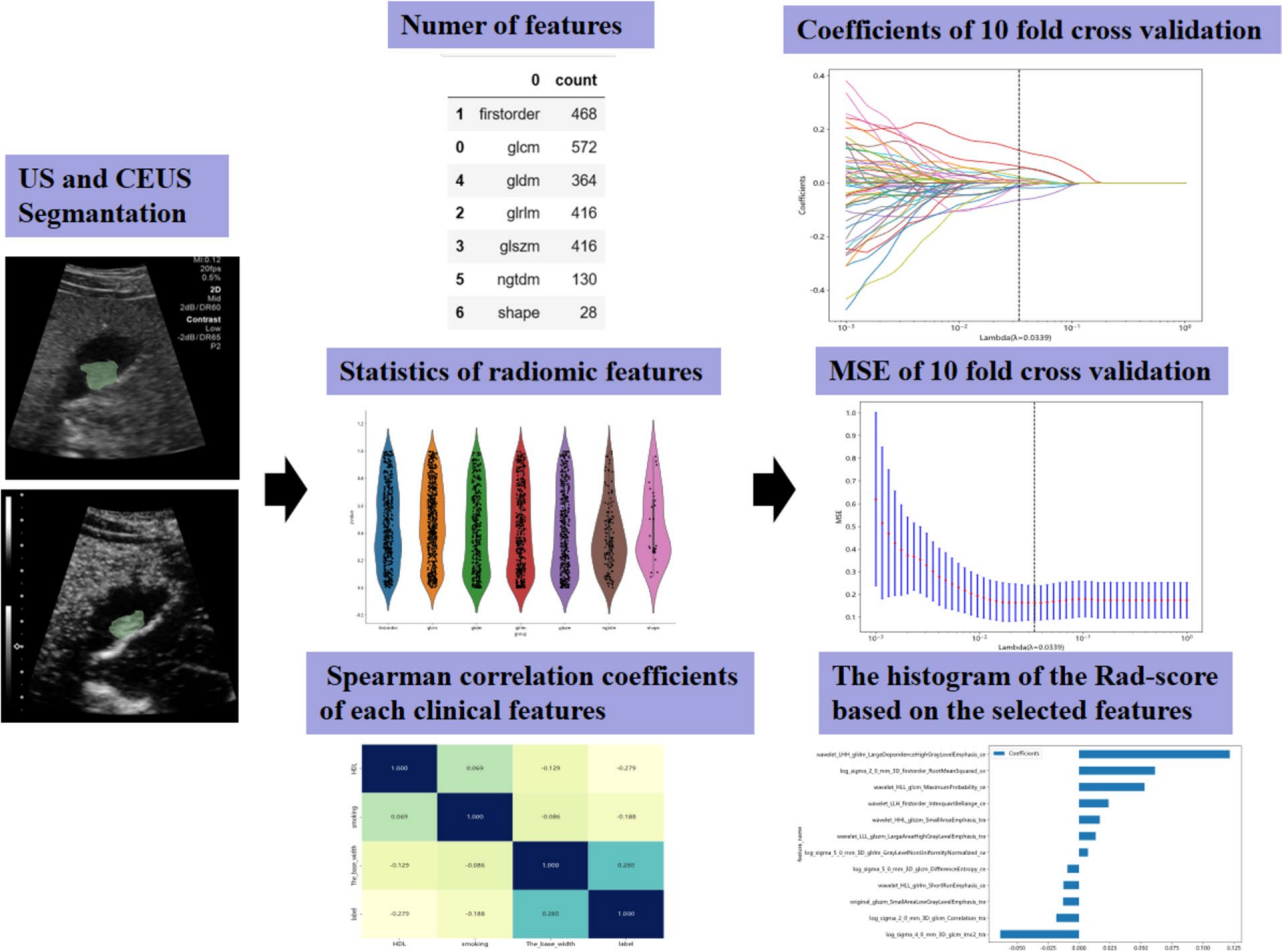
Flowchart of radiomics analysis process based on US and CEUS. US = conventional ultrasound, CEUS = contrast-enhanced ultrasound, MSE = Mean Squared Error; Rad-score, radiomics score

Divergences in scoring amongst observers, even for identical images encompassing those of targeted lesions, underwent meticulous review by a trio of ultrasound specialists to forge a consensus prior to initiating the segmentation of the region of interest (ROI). A pair of ultrasound physicians, utilizing the open-source 3D slicer V4.10.2 software (https://www.slicer.org/), delineated the ROIs at every targeted lesion (Fig. 2).

Three primary categories encompass the handcrafted features: (I) geometry, which delineates the three-dimensional form attributes of the tumor; (II) intensity, elucidating the primary-order statistical spread of voxel intensities within the neoplastic mass; and (III) texture, illustrating the configurations or second- and higher-order spatial dispersions of such intensities. Extracting texture features leverages various techniques, inclusive of the gray-level co-occurrence matrix (GLCM), gray-level run length matrix (GLRLM), gray-level size zone matrix (GLSZM), and neighborhood gray-tone difference matrix (NGTDM) approaches. The entirety of the tumor volume constituted the tumoral ROI (Fig. 2). Subsequent to ROI segmentation for each patient, a total of 5 ROIs were discerned from both US and CEUS. Employing 3D slicer software, complemented by an additional “PyRadiomics package” plug-in (https://www.radiomics.io/pyradiomics.html), extraction of radiomic features from each ROI was facilitated. This plug-in autonomously harvested a total of 1197 radiomic features from each individual ROI, with the pertinent radiomic data from each detailed extensively in Table 2.

### Feature selection and Rad signature

All radiomic features underwent scrutiny through the application of the Mann–Whitney U test and a thorough feature screening process. The strict retention criterion for a radiomic feature was a *P*-value below 0.05. Concurrently, Spearman's rank correlation coefficient took on the role of measuring the inter-feature correlation for those features demonstrating robust repeatability. When the correlation coefficient surpassed 0.9 between any two features, only one of the features was maintained. To maximize the representational capability of the features, a greedy recursive deletion strategy enacted feature filtering, successively omitting the feature exhibiting the most substantial redundancy within the present set. Subsequent to this methodology, US radiomic signatures, CEUS radiomic signatures, and the amalgamation of both signatures were condensed, preserving a total of ten features.

The method utilized for signature formulation on the discovery dataset employed the least absolute shrinkage and selection operator (LASSO) regression model. LASSO, contingent upon the regulatory weight λ, propels all regression coefficients towards zero, simultaneously assigning a zero value to the coefficients of numerous non-essential features. An optimal λ was identified by executing a tenfold cross-validation with minimum criteria, with the final λ value correlating to minimized cross-validation error. Subsequent to this, features retaining non-zero coefficients were utilized for regression model fitting, amalgamating into a single radiomics signature. A radiomics score for each individual patient was computed by orchestrating a linear combination of preserved features, each weighted by their respective model coefficients. For LASSO regression modeling, the Python scikit-learn package found utilization. Post LASSO feature screening, final features were channeled into various machine learning models such as SVM, LR, MLP, KNN, and XGBoost for the formulation of risk models. Employing a fivefold cross-verification strategy assisted in deriving the final Radiomics Signature (Fig. 2). With an aim to amplify the predictive efficiency of the model, a nomogram was constructed, amalgamating the Rad Signature with HDL, base width, and smoking data.

In addition, the incremental prognostic significance of the radiomics signature, when juxtaposed with clinical risk factors, was succinctly and effectively appraised via the exhibition of a radiomics nomogram within the validation data set framework. This particular nomogram integrated both the radiomics signature and pertinent clinical risk factors, founded upon the analytical framework provided by logistic regression (LR) analysis. To assess congruence between the neoplastic GBP prediction rendered by the nomogram and empirical observations, computation of the calibration curve ensued.

### Clinical signature

Clinical characteristics, encompassing age, gender, pathological diagnosis, and specific laboratory tests [inclusive of liver function, total cholesterol, CA199, and carcinoembryonic antigen (CEA)], underwent retrospective accumulation and notation for each patient. Initially, selection of features, intended for the construction of a clinical signature, derived from baseline statistics, ensuring *P*-value was maintained below 0.05. The identical machine learning model employed in the building process of the rad signature was utilized. Employing a fivefold cross-validation and establishing a test cohort remained constant, thereby ensuring a fair comparison was achieved.

### Radiomic nomogram

A radiomic nomogram was formulated by integrating both the radiomic signature and the clinical signature. Subsequent verification of the diagnostic efficacy of this radiomic nomogram was conducted within a test cohort, involving the deployment of Receiver Operating Characteristic (ROC) curves to scrutinize the diagnostic effectiveness of the nomogram. Calibration curves provided an evaluation of the nomogram's calibration efficiency, while the Hosmer–Lemeshow test facilitated an assessment of the nomogram's calibration capabilities. Additionally, a decision curve analysis (DCA) was mapped to assess the clinical utility of the predictive models under consideration.

### Statistical methods

Analytical procedures were executed utilizing IBM’s SPSS Statistics 28 software (SPSS Inc., Chicago, IL, USA) in conjunction with the R programming language, version 4.0.3 (R Foundation for Statistical Computing, Vienna, Austria; accessible at http://www.r-project.org/). Quantitative parameters found expression as means ± standard deviations (SDs). The comparative analysis of these quantitative parameters was conducted through the employment of either a t-test or a Mann–Whitney U test. Categories of variables were demarcated based upon clinical results, with their representation involving numerical counts and associated percentages. The ensuing statistical scrutiny of these variables was undertaken employing either Fisher’s exact test or the Chi-squared test. In the logistic regression (LR) model, all factors were encompassed, with only those variables displaying statistical significance (*P* < 0.05) being incorporated into a multivariate LR model through a sophisticated sequential strategy, which necessitated the computation of odds ratios (ORs). Noteworthy variables presented an Area Under the Curve (AUC) > 0.7. The efficacy of the nomogram was assessed utilizing the concordance index (c-index), with model validation being executed with the validation cohort and the assistance of R software.

## Results

### Clinical and ultrasonic characteristics of patients

An examination of the patients’ demographic details, Ultrasound (US) features, and Contrast-Enhanced Ultrasound (CEUS) characteristics across both training and test cohorts was conducted, as outlined in Table 1. The juxtaposition of multiple characteristics, including age, height, and weight, among the patient groups failed to reveal any statistical significance in the disparities (*P* > 0.05). Subsequent analyses incorporated specific indicators that exhibited notable differences. In our analysis of demographics and clinical characteristics, we found no significant differences in age and gender distribution between neoplastic and non-neoplastic gallbladder polyp groups (*P* > 0.05), suggesting that these basic parameters are insufficient for distinguishing between the two. However, HDL levels and basal widths showed significant differences (*P* < 0.05), indicating their potential as diagnostic indicators, yet they are not definitive on their own. Regarding imaging parameters, US measurements such as major axis, broad diameter, and basal width revealed significant differences (*P* < 0.05), but their individual diagnostic accuracy is limited. CEUS features, particularly vascular patterns and enhancement levels, exhibited more pronounced differences, highlighting their enhanced diagnostic value. To assess the diagnostic potential of these parameters, we calculated sensitivity, specificity, positive predictive value (PPV), and negative predictive value (NPV) for key features like basal width, enhancement mode, and vascular pattern. These calculations confirmed that individual features, while having some diagnostic value, fall short in accurately differentiating neoplastic from non-neoplastic polyps. The results will clearly show that individual parameters lack the sensitivity and specificity required for accurate diagnosis. This limitation underscores the necessity of combining these parameters with advanced radiomic features extracted from CEUS data, as our model achieves significantly higher diagnostic performance (AUC = 0.95 in internal validation).

**Table 1:** Baseline clinical and ultrasonic characteristics of patients in two cohorts

Characters	Training cohort				Test cohort			
	ALL (*n* = 83)	Non-NGB (*n* = 65)	NGB (*n* = 18)	*P* value	ALL (*n* = 36)	Non-NGB (*n* = 29)	NGB (*n* = 7)	*P* value
Age (y)	49.54 ± 10.99	49.37 ± 11.79	50.17 ± 7.73	0.991	50.14 ± 9.09	49.55 ± 9.10	52.57 ± 9.34	0.363
Heigth (cm)	164.28 ± 7.45	164.08 ± 6.88	165.00 ± 9.41	0.938	164.97 ± 6.56	164.55 ± 5.65	166.71 ± 9.86	0.967
Weight (kg)	65.26 ± 9.79	65.73 ± 9.35	63.56 ± 11.39	0.332	65.28 ± 10.37	64.43 ± 8.31	68.79 ± 16.95	0.836
Sex				0.869				0.848
M	47 (56.63%)	36 (55.38%)	11 (61.11%)		22 (61.11%)	17 (58.62%)	5 (71.43%)	
F	36 (43.37%)	29 (44.62%)	7 (38.89%)		14 (38.89%)	12 (41.38%)	2 (28.57%)	
Drinking				0.648				0.565
N	79 (95.18%)	61 (93.85%)	18 (100%)		31 (86.11%)	24 (82.76%)	7 (100%)	
Y	4 (4.82%)	4 (6.15%)	0 (0%)		5 (13.89%)	5 (17.24%)	0 (0%)	
Smoking				0.172				0.71
N	73 (87.95%)	55 (84.62%)	18 (100%)		32 (88.89%)	25 (86.21%)	7 (100%)	
Y	10 (12.05%)	10 (15.38%)	0 (0%)		4 (11.11%)	4 (13.79%)	0 (0%)	
DM				1.0				1.0
N	74 (89.16%)	58 (89.23%)	16 (88.89%)		34 (94.44%)	27 (93.10%)	7 (100%)	
Y	9 (10.84%)	7 (10.77%)	2 (11.11%)		2 (5.56%)	2 (6.9%)	0 (0%)	
Hightension				0.487				1.0
N	72 (86.75%)	55 (84.62%)	17 (94.44%)		33 (91.67%)	27 (93.10%)	6 (85.71%)	
Y	11 (13.25%)	10 (15.38%)	1 (5.56%)		3 (8.33%)	2 (6.90%)	1 (14.29%)	
Hyperlipemia				0.513				0.838
N	78 (93.98%)	60 (92.31%)	18 (100%)		34 (94.44%)	28 (96.55%)	6 (85.71%)	
Y	5 (6.02%)	5 (7.69%)	0 (0%)		2 (5.56%)	1 (3.45%)	1 (14.29%)	
Fattyliver				0.447				0.339
N	59 (71.08%)	48 (73.85%)	11 (61.11%)		28 (77.78%)	24 (82.76%)	4 (57.14%)	
Y	24 (28.92%)	17 (26.15%)	7 (38.89%)		8 (22.22%)	5 (17.24%)	3 (42.86%)	
Tumor history				0.41				0.52
N	77 (92.77%)	59 (90.77%)	18 (100.00%)		31 (86.11%)	26 (89.66%)	5 (71.43%)	
Y	6 (7.23%)	6 (9.23%)	0 (0%)		5 (13.89%)	3 (10.34%)	2 (28.57%)	
Neutrophil	3.58 ± 1.46	3.59 ± 1.29	3.58 ± 2.01	0.627	3.56 ± 0.91	3.60 ± 0.99	3.42 ± 0.46	0.146
Lymphocyte (10^9^/L)	1.82 ± 0.45	1.79 ± 0.40	1.91 ± 0.59	0.556	1.72 ± 0.47	1.67 ± 0.46	1.90 ± 0.54	0.3
Triglyceride (mmol/L)	1.60 ± 0.77	1.50 ± 0.48	1.96 ± 1.35	0.232	1.66 ± 0.91	1.50 ± 0.38	2.34 ± 1.88	0.237
HDL (mmol/L)	1.33 ± 0.29	1.39 ± 0.25	1.15 ± 0.35	0.006	1.32 ± 0.21	1.35 ± 0.18	1.21 ± 0.27	0.219
LDL (mmol/L)	3.05 ± 0.76	3.10 ± 0.64	2.89 ± 1.09	0.603	2.84 ± 0.51	2.82 ± 0.37	2.96 ± 0.92	0.339
Cholesterol (mmol/L)	4.62 ± 0.76	4.67 ± 0.62	4.45 ± 1.12	0.616	4.41 ± 0.56	4.33 ± 0.42	4.72 ± 0.91	0.682
Total bilirubin (mmol/L)	13.31 ± 5.30	13.47 ± 5.21	12.74 ± 5.74	0.542	13.36 ± 4.05	12.96 ± 3.61	15.01 ± 5.57	0.368
AST (mmol/L)	20.49 ± 8.35	20.43 ± 8.62	20.69 ± 7.53	0.702	21.35 ± 7.36	21.41 ± 7.47	21.07 ± 7.47	0.623
ALT (mmol/L)	24.78 ± 16.20	23.90 ± 14.25	27.95 ± 22.06	0.933	24.12 ± 13.70	24.49 ± 14.59	22.59 ± 9.91	0.917
Glucose (mmol/L)	5.22 ± 0.93	5.18 ± 0.85	5.36 ± 1.19	0.796	5.06 ± 0.61	5.00 ± 0.49	5.31 ± 0.96	0.95
CA199 (U/mL)	18.88 ± 52.93	20.61 ± 59.60	12.64 ± 9.54	0.573	16.25 ± 14.89	16.88 ± 16.41	13.63 ± 5.11	0.704
CEA (U/mL)	1.84 ± 1.11	1.86 ± 1.10	1.76 ± 1.15	0.309	1.81 ± 0.65	1.91 ± 0.66	1.41 ± 0.48	0.083
Number				0.31				0.742
Single	49 (59.04%)	36 (55.38%)	13 (72.22%)		20 (55.56%)	17 (58.62%)	3 (42.86%)	
Multiple	34 (40.96%)	29 (44.62%)	5 (27.78%)		16 (44.44%)	12 (41.38%)	4 (57.14%)	
Shape				0.225				0.235
Circle	4 (4.82%)	4 (6.15%)	0 (0%)		4 (11.11%)	4 (13.79%)	0 (0%)	
Ellipse	72 (86.75%)	57 (87.69%)	15 (83.33%)		27 (75.00%)	20 (68.97%)	7 (100.00%)	
Irregular	7 (8.43%)	4 (6.15%)	3 (16.67%)		5 (13.89%)	5 (17.24%)	0 (0%)	
Major axis (mm)	14.64 ± 7.08	13.32 ± 4.76	19.39 ± 11.23	0.005	13.36 ± 4.33	12.40 ± 2.67	17.37 ± 7.27	0.024
Broad diameter (mm)	9.44 ± 5.19	8.33 ± 2.81	13.42 ± 8.87	0.004	8.74 ± 4.19	7.80 ± 2.51	12.66 ± 7.13	0.046
Target lesion location				0.248				0.713
Base	31	26	5		17	14	3	
Neck	16	13	3		7	4	3	
Body	36	26	10		12	11	1	
The base width (mm)	4.73 ± 2.92	4.34 ± 2.59	6.13 ± 3.63	0.021	4.09 ± 1.24	3.90 ± 1.25	4.90 ± 0.87	0.023
Base				1.0				1.0
N	22 (26.51%)	17 (26.15%)	5 (27.78%)		13 (36.11%)	10 (34.48%)	3 (42.86%)	
Y	61 (73.49%)	48 (73.85%)	13 (72.22%)		23 (63.89%)	19 (65.52%)	4 (57.14%)	
Surface				0.011				0.165
Smooth	73 (87.95%)	60 (92.31%)	13 (72.22%)		27 (75.00%)	22 (75.86%)	5 (71.43%)	
Irregular	8 (9.64%)	3 (4.62%)	5 (27.78%)		5 (13.89%)	5 (17.24%)	0 (0%)	
Nodular	2 (2.41%)	2 (3.08%)	0 (0%)		4 (11.11%)	2 (6.90%)	2 (28.57%)	
Echo				0.777				0.141
Low	40 (48.19%)	30 (46.15%)	10 (55.56%)		17 (47.22%)	16 (55.17%)	1 (14.29%)	
Equal	38 (45.78%)	31 (47.69%)	7 (38.89%)		15 (41.67%)	10 (34.48%)	5 (71.43%)	
High	5 (6.02%)	4 (6.15%)	1 (5.56%)		4 (11.11%)	3 (10.34%)	1 (14.29%)	
Gallbladder_wall				1.0				1.0
N	73 (87.95%)	57 (87.69%)	16 (88.89%)		33 (91.67%)	27 (93.10%)	6 (85.71%)	
Y	10 (12.05%)	8 (12.31%)	2 (11.11%)		3 (8.33%)	2 (6.90%)	1 (14.29%)	
Calculus				0.276				1.0
N	73 (87.95%)	59 (90.77%)	14 (77.78%)		31 (86.11%)	25 (86.21%)	6 (85.71%)	
Y	10 (12.05%)	6 (9.23%)	4 (22.22%)		5 (13.89%)	4 (13.79%)	1 (14.29%)	
Cholecystitis				0.329				1.0
N	76 (91.57%)	58 (89.23%)	18 (100.00%)		33 (91.67%)	27 (93.10%)	6 (85.71%)	
Y	7 (8.43%)	7 (10.77%)	0 (0%)		3 (8.33%)	2 (6.90%)	1 (14.29%)	
Close to gallbladder_wall				0.218				1.0
Not complete	77 (92.77%)	62 (95.38%)	15 (83.33%)		33 (91.67%)	27 (93.10%)	6 (85.71%)	
Complete	6 (7.23%)	3 (4.62%)	3 (16.67%)		3 (8.33%)	2 (6.90%)	1 (14.29%)	
The base width of CEUS (mm)	5.82 ± 5.52	4.80 ± 2.07	9.52 ± 10.60	< 0.001	5.02 ± 2.01	4.80 ± 1.66	5.94 ± 3.08	0.582
CDFI				0.324				0.096
No	53 (63.86%)	43 (66.15%)	10 (55.56%)		26 (72.22%)	23 (79.31%)	3 (42.86%)	
Spot	20 (24.10%)	16 (24.62%)	4 (22.22%)		6 (16.67%)	3 (10.34%)	3 (42.86%)	
Strip	10 (12.05%)	6 (9.23%)	4 (22.22%)		4 (11.11%)	3 (10.34%)	1 (14.29%)	
Enhancement level				0.301				0.368
N	5 (6.02%)	4 (6.15%)	1 (5.56%)		4 (11.11%)	4 (13.79%)	0 (0%)	
Equal	31 (37.35%)	27 (41.54%)	4 (22.22%)		14 (38.89%)	12 (41.38%)	2 (28.57%)	
High	47 (56.63%)	34 (52.31%)	13 (72.22%)		18 (50.00%)	13 (44.83%)	5 (71.43%)	
Enhancement mode				0.228				0.134
Even	58 (69.88%)	48 (73.85%)	10 (55.56%)		25 (69.44%)	18 (62.07%)	7 (100.00%)	
Uneven	25 (30.12%)	17 (26.15%)	8 (44.44%)		11 (30.56%)	11 (37.93%)	0 (0%)	
Vascular level				< 0.001				0.811
Irregular	8 (9.64%)	5 (7.69%)	3 (16.67%)		5 (13.89%)	4 (13.79%)	1 (14.29%)	
Dot	50 (60.24%)	46 (70.77%)	4 (22.22%)		14 (38.89%)	12 (41.38%)	2 (28.57%)	
Branch	25 (30.12%)	14 (21.54%)	11 (61.11%)		17 (47.22%)	13 (44.83%)	4 (57.14%)	

NGB = neoplastic GBP, M = man, F = female, *N* = no, Y = yes, HDL = High-density lipoprotein, LDL = Low-density lipoprotein, ALT = alanine aminotransferase, AST = aspartate aminotransferase, CEUS = contrast-enhanced ultrasound, CDFI = color doppler flow imaging, *P* value for comparisons between non-NGB and NGB patients

### Features statistics and LASSO feature selection

Extraction of 1197 × 2 handcrafted features spanned across nine distinct categories, which included 18 × 2 first-order features and 14 shape features, with the remainder categorized as texture features. Utilization of an in-house feature analysis program, implemented via Pyradiomics (http://pyradiomics.readthedocs.io), facilitated the extraction of all handcrafted features. An analysis of the statistical data of radiomic features presents a comprehensive overview of all features, alongside their respective *P*-value results, comprehensively presented in Table 2. An exploration utilizing Spearman correlation coefficients among each clinical feature underscores correlations, revealing that Long Diameter, Short Diameter, and Diameter possess the highest correlation coefficient.

**Table 2:** The results of radiomics feature selection based on cross-validation

Sequence	Feature number	Individual features
US	10	log_sigma_2_0_mm_3D_glcm_Correlation log_sigma_2_0_mm_3D_gldm_DependenceEntropy log_sigma_3_0_mm_3D_glrlm_RunEntropy log_sigma_4_0_mm_3D_glcm_Imc2 original_glszm_SmallAreaLowGrayLevelEmphasis wavelet_HHH_firstorder_Median wavelet_HHH_glcm_Autocorrelation wavelet_HHL_glszm_SmallAreaEmphasis wavelet_HLL_glszm_ZoneVariance wavelet_LLL_glszm_LargeAreaHighGrayLevelEmphasis
CEUS	10	log_sigma_2_0_mm_3D_firstorder_RootMeanSquare log_sigma_4_0_mm_3D_glcm_Imc2 log_sigma_5_0_mm_3D_glcm_DifferenceEntropy log_sigma_5_0_mm_3D_glrlm_GrayLevelNonUniformityNormalized wavelet_HLL_firstorder_Mean wavelet_HLL_glcm_MaximumProbability wavelet_HLL_glrlm_ShortRunEmphasis_ce wavelet_LHH_gldm_LargeDependenceHighGrayLevelEmphasis wavelet_LHL_ngtdm_Contrast wavelet_LLH_firstorder_InterquartileRange
US + CEUS	10	log_sigma_2_0_mm_3D_glcm_Correlation_tra log_sigma_4_0_mm_3D_glcm_Imc2_tra original_glszm_SmallAreaLowGrayLevelEmphasis_tra wavelet_HHL_glszm_SmallAreaEmphasis_tra wavelet_LLL_glszm_LargeAreaHighGrayLevelEmphasis_tra log_sigma_2_0_mm_3D_glcm_Correlation_tra log_sigma_4_0_mm_3D_glcm_Imc2_tra original_glszm_SmallAreaLowGrayLevelEmphasis_tra wavelet_HHL_glszm_SmallAreaEmphasis_tra wavelet_LLL_glszm_LargeAreaHighGrayLevelEmphasis_tra

US = Conventional ultrasound, CEUS = contrast-enhanced ultrasound

For the establishment of the Rad-score, nonzero coefficients were identified and utilized, deploying a least absolute shrinkage and selection operator (LASSO) logistic regression model. An exhibition of coefficients and the Mean Standard Error (MSE) from a 10-folds validation is depicted in Fig. 2. Furthermore, the histogram of the Rad-score, grounded on the chosen features, illustrates the coefficient values within the final selection of non-zero features.

### Univariate and multivariate analyses of clinical and ultrasonic characteristics

**Fig. 3:**
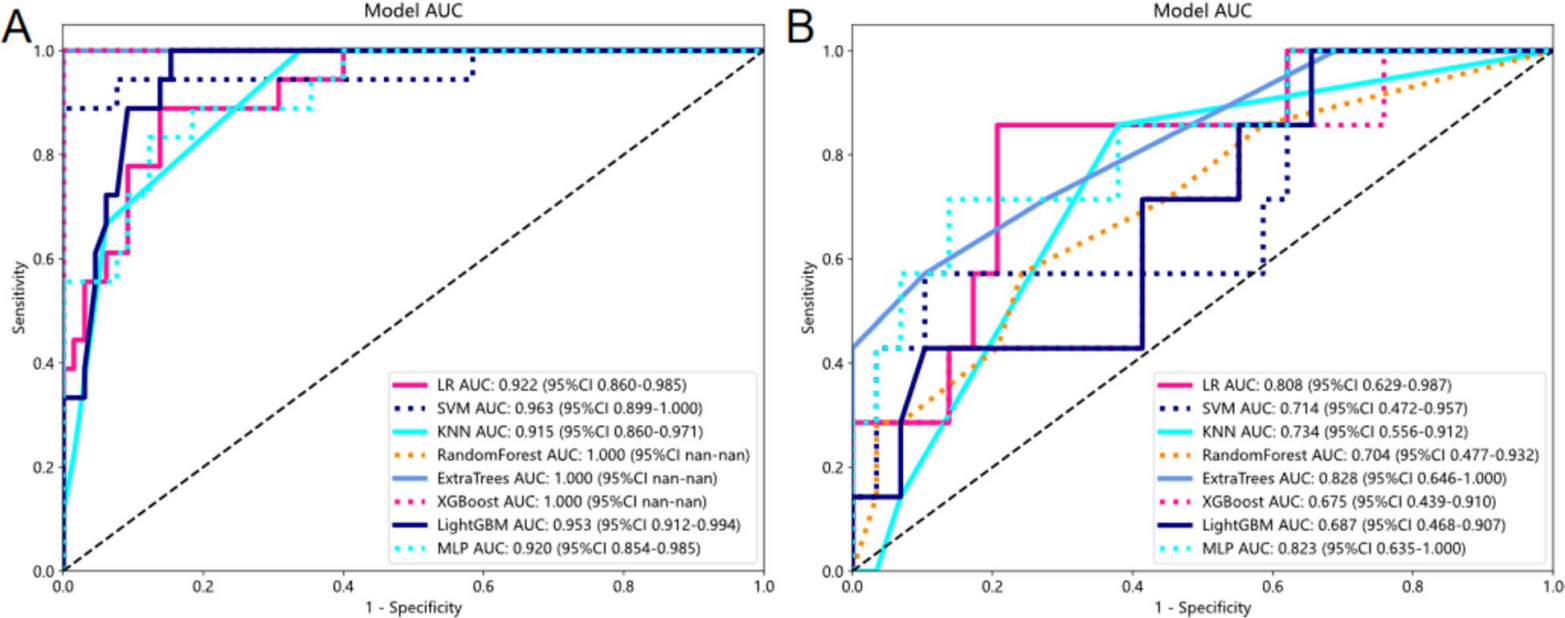
ROC analysis of different models on rad signature: the training cohort **(A)**; the test cohort **(B)**

Within the training cohort, the univariate analysis illuminated associations that held statistical significance with HDL (high-density lipoprotein) (*P* = 0.002), the major axis (*P* = 0.001), broad diameter (*P* < 0.001), the foundational width (*P* = 0.021), the foundational width as per CEUS (*P* = 0.001), and Triglyceride (*P* = 0.024). In a subsequent layer of analysis, the multivariate approach determined HDL (*P* = 0.04) as the sole variable manifesting statistically significant associations, as detailed in Table S1. An analytical approach involving several deep learning algorithms was adopted to scrutinize the performance of clinical signatures, the details of which are tabulated in Table S2. Tables S3 and S4 encompass all models employed to predict neoplastic GBP, with the LR model showcasing the pinnacle of performance. Thus, in the constitution of ultrasonic signatures, LR was chosen as the foundational model. Comparative analysis utilizing rad features facilitated the derivation of the optimal model when juxtaposed with a KNN, ExtraTrees, XGBoost, and LightGBM classifier. The model, comprising US coupled with CEUS, accomplished the highest AUC value in both the training and test cohort, registering 0.922 and 0.808 respectively, in the prediction of neoplastic GBP (Table S5). Figure 3 delineates the AUC of each rad signature model within the training and test cohorts. The AUC for CEUS radiomic signatures was 0.89 (95% CI: 0.82–0.97), outperforming US radiomic signatures with an AUC of 0.85 (95% CI: 0.75–0.94). Supplementary Table 5 shows that the combined use of conventional ultrasound and CEUS radiomic signatures resulted in an AUC of 0.922 (95% CI: 0.86—0.98) for conventional ultrasound features, indicating an improved diagnostic performance when both modalities are used together.

To compare the performance of the radiomics model with machine learning models, we employed various machine learning algorithms, including SVM, LR, MLP, KNN, and XGBoost. These algorithms were chosen for their diverse approaches to handling complex datasets and their established effectiveness in medical diagnostic predictions. As detailed in Supplementary Table 2, we utilized various machine learning models based on clinical information to predict malignant gallbladder polyps. Supplementary Table 3 displays the predictions made using only conventional ultrasound information, while Supplementary Table 4 focuses on predictions derived from CEUS information. By integrating both conventional ultrasound and CEUS data, Supplementary Table 5 demonstrates the enhanced predictive capability of the combined radiomics model, with an AUC of 0.922.

### Performance of the radiomics models

**Fig. 4:**
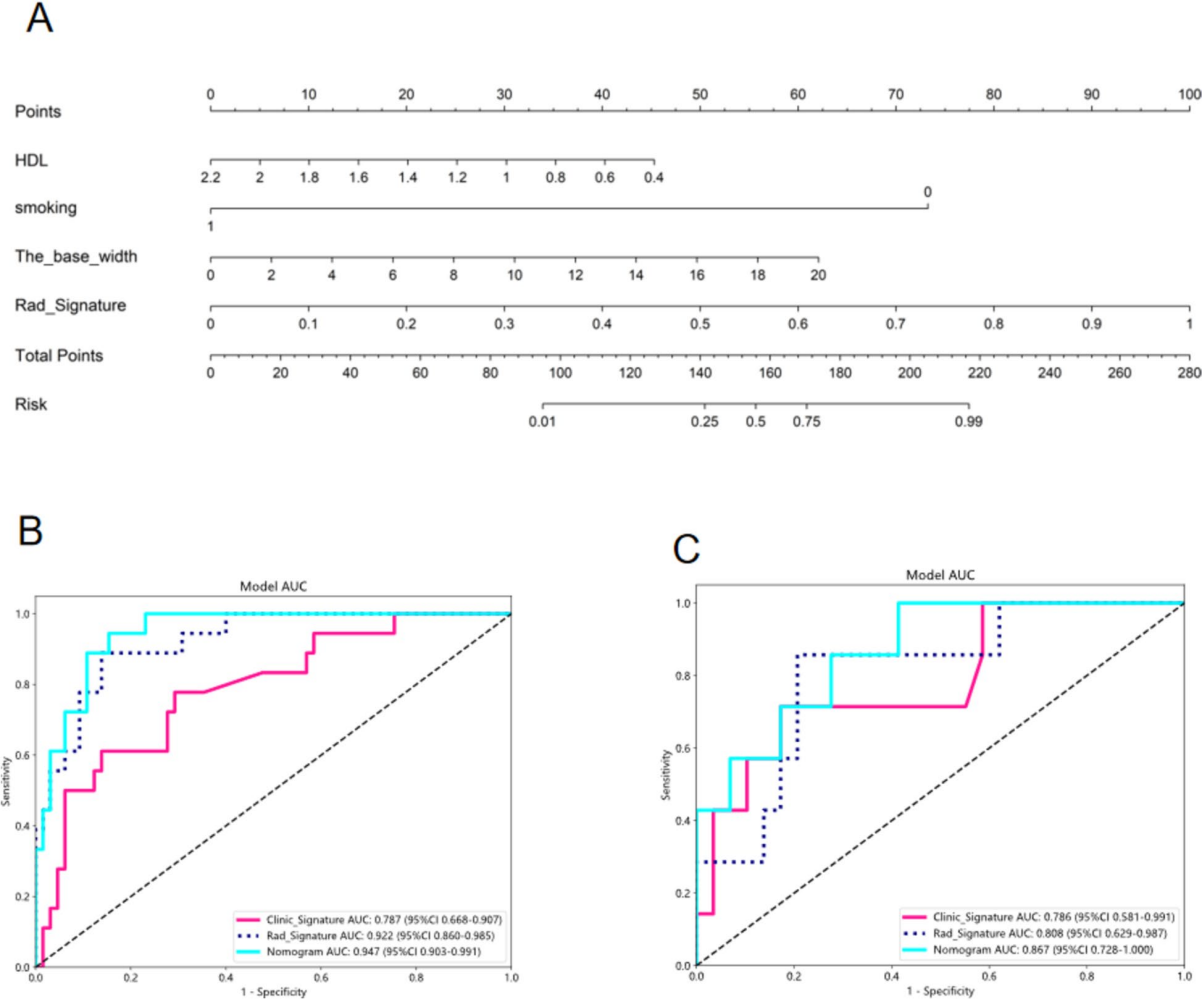
The nomogram for clinical use **(A)** show the AUC in both train **(B)** and test cohort** (C)**

**Fig. 5:**
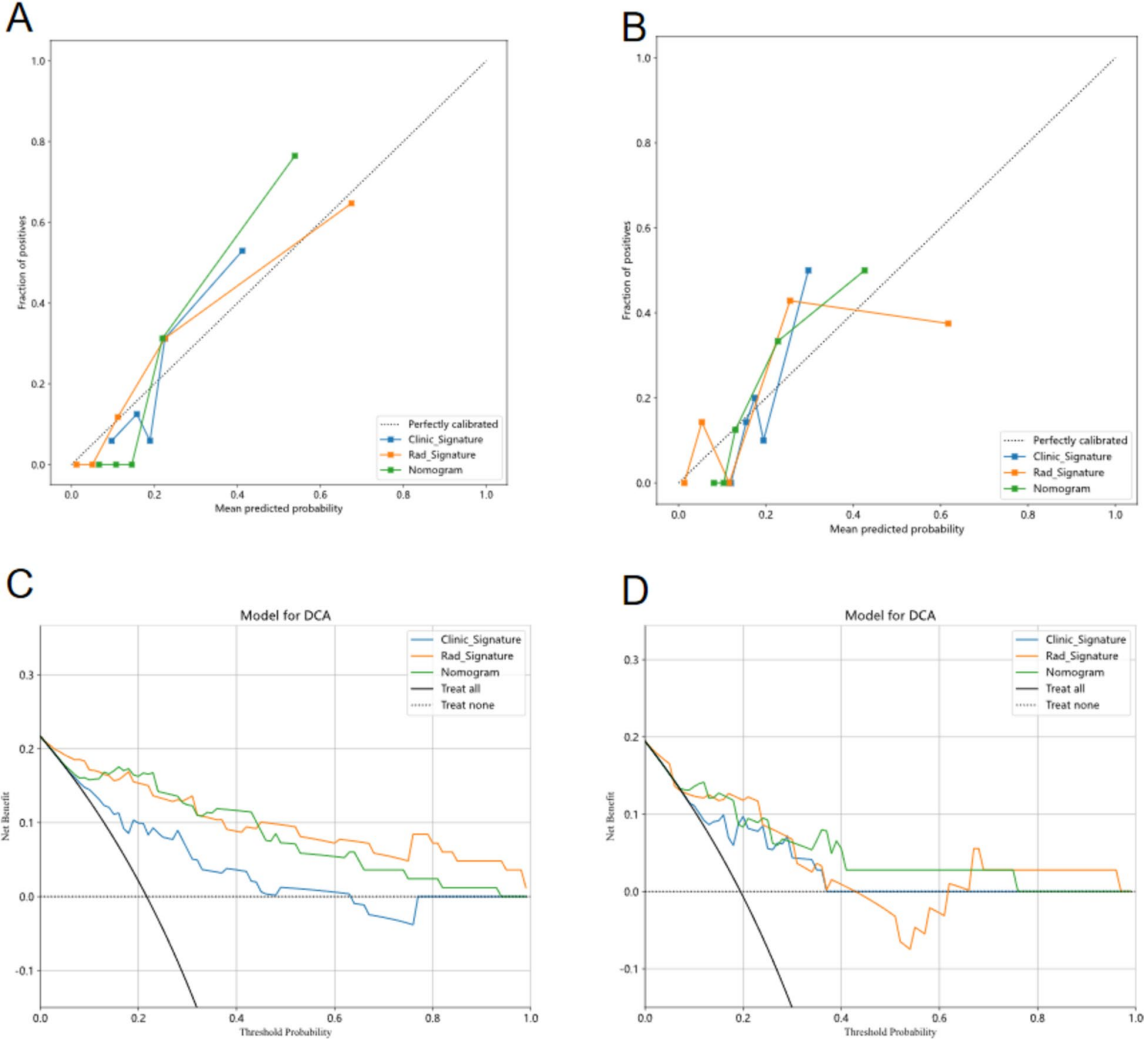
The calibration curves in train and test cohort: Hosmer–Lemeshow test **(A, B)** Decision curve of in test cohort** (C, D)**

In the training cohort, exemplary fitting was observed for both clinical and rad signatures. Conversely, in the test cohort, the clinical signature demonstrated a tendency towards overfitting, while the rad signature maintained a satisfactory fit. Employing the LR algorithm, a Nomogram was constructed, amalgamating clinical and rad signatures, and exhibited optimal performance, as visualized in Fig. 4. A comparative analysis between the clinical signature, rad signature, and nomogram was conducted and is presented in Table 3, with the Delong test utilized for statistical assessment in Table 4. Nomogram calibration curves affirmed a commendable congruence between the predicted and observed neoplastic GBP within both training and test cohorts. The application of the Hosmer–Lemeshow test inspected the fit of clinical signature, rad signature, and nomogram. Analysis of the Clinic Signature through the Hosmer–Lemeshow test for training and test cohorts resulted in P values of 0.163 and 0.106, respectively. Moreover, the Rad Signature, when subjected to the same test, provided P values of 0.933 and 0.233 for training and validation cohorts, respectively. Furthermore, application of the Hosmer–Lemeshow test to the Nomogram yielded P values of 0.551 and 0.544 for training and validation cohorts, respectively, indicating an impeccable fit for both. Calibration curves for train and test cohorts are delineated in Fig. 5A, B. Moreover, each model underwent evaluation through Decision Curve Analysis (DCA). Figure 5C, D illustrates the decision curve analysis for the clinical signature, rad signature, and radiomic nomogram. The radiomic nomogram indicated a considerable benefit for interventions in patients, particularly when prediction probability was juxtaposed with clinical signature and rad signature, and exceeded other signatures. The utility of the radiomic nomogram for the preoperative prediction of neoplastic GBP has demonstrated enhanced clinical advantage.

**Table 3:** Comparison of clinic signature, Rad Signature and the nomogram

Signature	Accuracy	AUC	95% CI	Sensitivity	Specificity	PPV	NPV	Precision	Recall	F1	Threshold
Clinic Signature-training	0.723	0.787	0.6677–0.9066	0.778	0.719	0.424	0.920	0.424	0.778	0.549	0.200
Rad Signature-training	0.867	0.922	0.8598–0.9846	0.889	0.862	0.640	0.966	0.640	0.889	0.744	0.188
Nomogram -training	0.867	0.947	0.9026–0.9914	0.944	0.846	0.630	0.982	0.630	0.944	0.756	0.187
Clinic Signature-test	0.806	0.786	0.5807–0.9907	0.714	0.828	0.500	0.923	0.500	0.714	0.588	0.202
Rad Signature-test	0.806	0.808	0.6288–0.9869	0.857	0.793	0.500	0.958	0.500	0.857	0.632	0.233
Nomogram-test	0.667	0.867	0.7282–1.0000	1.000	0.586	0.368	1.000	0.368	1.000	0.538	0.127

Rad Signature = conventional ultrasound combined with contrast-enhanced ultrasound radiomic signatures, nomogram = the combined model of clinic signatures, conventional ultrasound and contrast-enhanced ultrasound radiomic signatures, AUC = Area Under Curve, CI = confidence interval, PPV = positive predictive value, NPV = negative predictive value

**Table 4:** The DELONG test was assessed the nomogram compared to the clinic signature and the nomogram compared to the Rad Signature

Cohort	Nomogram vs clinic	Nomogram vs rad
Training	0.002	0.430
Test	0.466	0.167

Rad Signature = conventional ultrasound combined with contrast-enhanced ultrasound radiomic signatures, nomogram = the combined model of clinic signatures, conventional ultrasound and contrast-enhanced ultrasound radiomic signatures

## Discussion

In this investigation, distinct analyses were conducted on laboratory assessments, US radiomic signatures, and CEUS radiomic signatures. Deep learning techniques facilitated the identification of optimal factors for modeling, leading to the establishment of an LR model. Exhibiting a potent predictive capacity, this model adeptly preoperatively prognosticates neoplastic GBP exceeding 10 mm. It was observed that CEUS radiomic signatures bore substantial weight in the model, aligning with prior investigations underscoring the pivotal role of CEUS in GBP diagnosis [24]. This approach not only mitigates subjective errors linked to gallbladder CEUS interpretation but also bridges an existing gap related to unexplored gallbladder CEUS radiomic signatures. Through the utilization of contrast agents, CEUS enhances image contrast and precision over conventional ultrasound, amplifying GBP visibility and facilitating a more nuanced differentiation from adjacent tissues to assist medical professionals in accurately diagnosing GBP. Consequently, CEUS finds extensive application in GBP diagnosis. Earlier research signified that tumor size emerges as a pivotal parameter in discerning GBP nature, with a notable positive correlation being observable in tumor-associated polyps relative to diameter [11]. This investigation corroborated that the basal width of the polyps stands as a discerning factor for tumor-related and non-tumor-related GBP, which is in agreement with preceding studies that found tumor-related polyps typically manifest with broader bases [25, 26]. In the training set, we observed statistically significant differences in base widths between non-NGB (4.34 ± 2.59) and NGB (6.13 ± 3.63), with a *P*-value of 0.021. Similarly, in the validation set, the base width for non-NGB was 3.90 ± 1.25, and for NGB it was 4.90 ± 0.87, yielding a *P*-value of 0.023, indicating statistical significance in both cases. These results align well with the updated joint guidelines [27], which emphasize the importance of polyp size as a critical factor in the management of gallbladder polyps. According to these guidelines, cholecystectomy is recommended for patients with polypoid lesions measuring 10 mm or more, which is consistent with our findings that larger base widths are associated with a higher risk of malignancy. The SRU consensus conference recommendations [28] also support a less aggressive approach to gallbladder polyp management, advocating for standardized evaluation by radiologists. Our study's findings, particularly the statistically significant differences in base widths, underscore the value of careful monitoring and the potential need for surgical intervention in line with these recommendations. The expansive base of neoplastic GBP might be attributed to its invasive character, where the invading tumor cells penetrate the deeper tissues of the gallbladder wall, expanding the polyp’s base and subsequently culminating in a broader base within the gallbladder wall. An independent risk factor, HDL was identified to have considerable influence in both univariate and multivariate logistic regression, aligning with previous findings [29]. The study noted that tumor-associated polyps exhibited diminished HDL levels, with patients demonstrating reduced HDL levels encountering a 3.035-fold heightened risk of malignant tumor development [30]. This phenomenon might be attributed to the aberrant proliferation and metabolic activity in tumor cells, potentially instigating localized lipid metabolic disturbances, which include HDL reduction. Moreover, the genesis of tumor-related GBP might also be interlinked with inflammatory responses [13], capable of modifying lipid metabolism, inclusive of the synthesis and conveyance of HDL, thereby catalyzing a decrement in HDL levels.

A palpable challenge emanating from the unavailability of histopathological tissue has been underscored by antecedent research, revealing the inherent difficulty in the preoperative differentiation between tumor-related GBP and non-tumor-related polyps. Specifically for GBP measuring in excess of 10 mm, surgical excision is frequently espoused for those polyps correlated with tumors. Nevertheless, considering the relatively minimal proportion of tumor-related polyps within the totality of GBP, there exists a propensity for inadvertently removing cholesterol polyps during gallbladder procedures [31]. In the pursuit of discerning benign from malignant GBP, a model melding both radiomics and clinical signatures manifested an AUC of 0.950, highlighting a potentially potent diagnostic tool [32]. Furthermore, a model conceptualized as a columnar chart, synergizing CT and inflammatory markers, revealed commendable efficacy in both the differentiation and prognostication of gallbladder polyp-like lesions, boasting an AUC of 0.964, in conjunction with 82.4% sensitivity and 97.8% specificity [13]. The Bayesian network model, anchored on singular polyps, polyp CSA ≥ 85 mm^2^, a fundus exhibiting a wide base, and median echogenicity, recorded accuracies of 81.88% and 82.35% for the training and test sets, respectively [33]. A further columnar chart model, predicated on clinical and ultrasound imaging biomarkers, exhibited proficient identification capabilities in both the training (AUC = 0.865) and validation (AUC = 0.845) groups [34]. However, a conspicuous scarcity is observed in the application of CEUS radiomic signatures as predictive elements within these models.

Ultrasound emerges as a pivotal tool during the preoperative evaluation of Gallbladder Polyps (GBP). Specifically, Contrast-Enhanced Ultrasound (CEUS), serving as a real-time observational modality, empowers medical practitioners to directly witness alterations and dynamic attributes of GBP throughout the examination process. This becomes instrumental in evaluating the polyps' blood flow, internal composition, and activity [35]. Furthermore, CEUS possesses the capacity to furnish multidimensional visuals, including three-dimensional or four-dimensional ultrasound imaging [36]. These visuals, in turn, afford a more exhaustive insight into the polyps’ morphology and localization, thereby facilitating physicians in ascertaining the GBP's nature and attributes with precision. Utilizing contrast agents, ultrasound contrast imaging notably enhances the contrast and resolution of images. In the conducted research, a comparison between US radiomic signatures and CEUS radiomic signatures elucidated that the latter (AUC = 0.848, 95%CI = 0.7548–0.9409) showcased superior predictive performance over the former (AUC = 0.892, 95% CI 0.8152–0.9694) (Table S2, S3). Consequently, a combination of US and CEUS radiomic signatures was chosen, which notably augmented the predictive performance (AUC = 0.922, 95% CI: 0.8598–0.9846), when juxtaposed with employing CEUS radiomic signatures independently. Fei et al.'s study highlights the advantages of H-CEUS in improving temporal resolution and accurately reflecting the microcirculation differences in GPLs, which is of great significance for selecting treatment plans [37]. Kim et al.'s study demonstrates the potential of deep learning in enhancing the accuracy of gallbladder polyp classification from ultrasound images, especially when combined with patient age and polyp size (38). Both studies indicate that a single diagnostic tool or parameter is often insufficient to provide adequate diagnostic information; their application in clinical practice requires the integration of other clinical parameters and imaging characteristics. This comprehensive diagnostic approach can improve diagnostic accuracy, reduce unnecessary cholecystectomies, and provide better treatment options for patients. Historically, seldom have studies amalgamated radiomics with deep learning to bolster the model's validity. Therefore, in a bid to integrate clinical indicators further with radiomic signatures, deep learning methodologies were employed, and the LR model was selected to construct a nomogram. The model evinced a robust predictive value (AUC = 0.947, 95% CI: 0.9026–0.9914), complemented by a sensitivity of 0.944 and specificity of 0.846 (Table 3). As shown in Table 3, the clinical signature, which includes ultrasound characteristics such as the maximum diameter of the lesion (Major_axis), the base width (The base width), blood flow (CDFI), and target lesion location, demonstrated a predictive AUC of 0.787 (95% Confidence Interval 0.668–0.907). The Radiomics Signature, derived from the radiomics analysis process based on US and CEUS, showed a superior AUC of 0.922 (95% CI 0.860–0.985). Furthermore, the Nomogram, which integrates the Radiomics Signature with clinical indicators, exhibited an AUC of 0.947 (95% CI: 0.903–0.991). These results substantiate that radiomics outperforms traditional ultrasound characteristics in diagnostic performance, and their combination yields optimal diagnostic efficacy. Hence, CEUS not only excels in diagnosing GBP’s nature but can also synergize with CEUS radiomic signatures to formulate models for GBP’s diagnosis, therapeutic efficacy evaluation, and prognostication. Despite these findings, the study encompasses limitations, such as being retrospective in nature, possessing a comparatively diminutive sample size, and necessitating validation through a more extensive sample. Moreover, the absence of multicenter data poses an additional limitation. We are committed to addressing it in future research endeavors. To bolster the robustness of our findings and enhance the generalizability of our results, we plan to expand our dataset by incorporating a larger and more diverse cohort of patients. This will allow us to more accurately assess the performance of our predictive model and to fine-tune our machine learning approach to better serve clinical decision-making in the management of gallbladder polyps. Our future work will prioritize data collection from multiple centers, ensuring a broader representation and leading to more comprehensive and impactful conclusions.

In summary, this research resulted in the formulation of a LR model, amalgamating clinical variables with ultrasonic radiomic signatures, designed to provide proficient preoperative diagnostic insight into neoplastic GBP of dimensions exceeding 10 mm. When the gallbladder mass was deemed benign, such as non-neoplastic polyps without notable symptoms, follow-up observations were chosen, obviating immediate surgery. In cases of suspected malignancy, to prevent rapid deterioration, prompt treatment was advised, with a rational treatment plan formulated. The model not only demonstrated elevated predictive prowess but also exhibited exemplary predictive value when applied to the preoperative diagnosis of such GBP entities. A key advantage rests in the potential of this model to preclude unnecessary gallbladder excisions in instances of cholesterol polyps while simultaneously minimizing the subjective evaluation errors traditionally associated with CEUS. Thus, the proposed model holds substantial promise for comprehensive application and widespread dissemination in the medical community.

## Supplementary Information

Additional file 1

## Data Availability

No datasets were generated or analysed during the current study.

## Abbreviations

GBP: Gallbladder polyps
US: Conventional ultrasound
CEUS: Contrast-enhanced ultrasound
SVM: Support vector machine
LR: Logistic regression
MLP: Multilayer perceptron
KNN: K-nearest neighbors
XGBoost: EXtreme gradient boosting
AUC: Area under the receiver operating characteristic curve
CI: Confidence interval

## References

[1] Myers RP, Shaffer EA, Beck PL. Gallbladder polyps: epidemiology, natural history and management. Can J Gastroenterol. 2002;16(3):187–94. 11930198 10.1155/2002/787598

[2] Kim KH. Gallbladder polyps: evolving approach to the diagnosis and management. Yeungnam Univ J Med. 2021;38(1):1–9. 33045805 10.12701/yujm.2020.00213 PMC7787897

[3] Son JH. Recent updates on management and follow-up of gallbladder polyps. Korean J Gastroenterol. 2023;81(5):197–202. 37226819 10.4166/kjg.2023.038 PMC12285348

[4] Park JY, Hong SP, Kim YJ, et al. Long-term follow up of gallbladder polyps. J Gastroenterol Hepatol. 2009;24:219–22. 19054258 10.1111/j.1440-1746.2008.05689.x

[5] Kalbi DP, Bapatla A, Chaudhary AJ, Bashar S, Iqbal S. Surveillance of gallbladder polyps: a literature review. Cureus. 2021;13(7): e16113. 34350077 10.7759/cureus.16113 PMC8325965

[6] Wennmacker SZ, Lamberts MP, Di Martino M, et al. Transabdominal ultrasound and endoscopic ultrasound for diagnosis of gallbladder polyps. Cochrane Database Syst Rev. 2018;8:CD012233. 30109701 10.1002/14651858.CD012233.pub2 PMC6513652

[7] Cocco G, Basilico R, Delli Pizzi A, et al. Gallbladder polyps ultrasound: what the sonographer needs to know. J Ultrasound. 2021;24:131–42. 33548050 10.1007/s40477-021-00563-1 PMC8137797

[8] van Dooren M, de Reuver PR. Gallbladder polyps and the challenge of distinguishing benign lesions from cancer. United Eur Gastroenterol J. 2022;10:625–6. 10.1002/ueg2.12287 PMC9486489 35951350

[9] Costa AG, Guerrero VL, Monforte MNG, et al. Is ultrasonography accurate for the diagnosis of gallbladder polyps? A review of cholecystectomy specimens from patients diagnosed with gallbladder polyps over a 14-years period. Cir Esp (Engl Ed). 2023;101:701–7. 37748643 10.1016/j.cireng.2023.02.009

[10] Chavan S, Rathi P. Gallbladder polyp: review and proposed algorithm for management. J Assoc Phys India. 2022;70:11–2. 35062812

[11] Fujiwara K, Abe A, Masatsugu T, Hirano T, Sada M. Effect of gallbladder polyp size on the prediction and detection of gallbladder cancer. Surg Endosc. 2021;35(9):5179–85. 32974780 10.1007/s00464-020-08010-8

[12] Donald G, Sunjaya D, Donahue T, et al. Polyp on ultrasound: now what? The association between gallbladder polyps and cancer. Am Surg. 2013;79:1005–8. 24160788

[13] Zhang J, Wu Y, Feng Y, Fu J, Jia N. The value of CT findings combined with inflammatory indicators for preoperative differentiation of benign and malignant gallbladder polypoid lesions. World J Surg Oncol. 2023;21(1):51. 36803518 10.1186/s12957-023-02941-x PMC9938612

[14] Chavan S, Rathi P. Gallbladder polyp: review and proposed algorithm for management. J Assoc Physicians India. 2022;70(1):11–2. 35062812

[15] Yang X, Liu Y, Guo Y, Chai R, Niu M, Xu K. Utility of radiomics based on contrast-enhanced CT and clinical data in the differentiation of benign and malignant gallbladder polypoid lesions. Abdom Radiol (NY). 2020;45(8):2449–58. 32166337 10.1007/s00261-020-02461-2

[16] Miwa H, Numata K, Sugimori K, Kaneko T, Maeda S. Vascular evaluation using transabdominal ultrasound for gallbladder polyps. J Med Ultrason (2001). 2021;48(2):159–73. 32125576 10.1007/s10396-020-01008-8

[17] Mayerhoefer ME, Materka A, Langs G, Häggström I, Szczypiński P, Gibbs P, Cook G. Introduction to radiomics. J Nucl Med. 2020;61(4):488–95. 32060219 10.2967/jnumed.118.222893 PMC9374044

[18] Yuan HX, Wang C, Tang CY, You QQ, Zhang Q, Wang WP. Differential diagnosis of gallbladder neoplastic polyps and cholesterol polyps with radiomics of dual modal ultrasound: a pilot study. BMC Med Imaging. 2023;23(1):26. 36747143 10.1186/s12880-023-00982-y PMC9901123

[19] Liao Z, Song Y, Ren S, Song X, Fan X, Liao Z. VOC-DL: Deep learning prediction model for COVID-19 based on VOC virus variants. Comput Methods Programs Biomed. 2022;224: 106981. 35863125 10.1016/j.cmpb.2022.106981 PMC9242688

[20] Zhu L, Han P, Lee R, Jiang B, Jiao Z, Li N, Tang W, Fei X. Contrast-enhanced ultrasound to assess gallbladder polyps. Clin Imaging. 2021;78:8–13. 33706069 10.1016/j.clinimag.2021.02.015

[21] Miwa H, Numata K, Sugimori K, Sanga K, Hirotani A, Tezuka S, Goda Y, Irie K, Ishii T, Kaneko T, Tanaka K, Maeda S. Differential diagnosis of gallbladder polypoid lesions using contrast-enhanced ultrasound. Abdom Radiol (NY). 2019;44(4):1367–78. 30478647 10.1007/s00261-018-1833-4

[22] Zhang HP, Bai M, Gu JY, He YQ, Qiao XH, Du LF. Value of contrast-enhanced ultrasound in the differential diagnosis of gallbladder lesion. World J Gastroenterol. 2018;24(6):744–51. 29456413 10.3748/wjg.v24.i6.744 PMC5807677

[23] Dietrich CF, Nolsøe CP, Barr RG, Berzigotti A, Burns PN, Cantisani V, et al. Guidelines and Good Clinical Practice Recommendations for Contrast Enhanced Ultrasound (CEUS) in the Liver – Update 2020 – WFUMB in Cooperation with EFSUMB AFSUMB AIUM and FLAUS. Abstract Ultraschall in der Medizin - Eur J Ultrasound 2020;41(05):562–85. 10.1055/a-1177-0530 10.1055/a-1177-0530 32707595

[24] Li Y, Tejirian T, Collins JC. Gallbladder polyps: real or imagined? Am Surg. 2018;84(10):1670–4. 30747692

[25] Tian F, Ma YX, Liu YF, Liu W, Hong T, He XD, Qu Q. Management strategy for gallbladder polypoid lesions: results of a 5-year single-center cohort study. Dig Surg. 2022;39(5–6):263–73. 36696883 10.1159/000529221

[26] Zhang D, Li Q, Zhang X, Jia P, Wang X, Geng X, Zhang Y, Li J, Yao C, Liu Y, Guo Z, Yang R, Lei D, Yang C, Hao Q, Yang W, Geng Z. Establishment of a nomogramprediction model for long diameter 10–15 mm gallbladder polyps with malignanttendency. Surgery. 2021;170(3):664–72. 34090677 10.1016/j.surg.2021.04.035

[27] Foley KG, Lahaye MJ, Thoeni RF, et al. Management and follow-up of gallbladder polyps: updated joint guidelines between the ESGAR, EAES. EFISDS and ESGE Eur Radiol. 2022;32:3358–68. 34918177 10.1007/s00330-021-08384-w PMC9038818

[28] Anderson MA, Mercaldo S, Cao J, et al. Society of radiologists in ultrasound consensus conference recommendations for incidental gallbladder polyp management: interreader agreement among 10 radiologists. AJR Am J Roentgenol. 2024;222: e2330720. 38353447 10.2214/AJR.23.30720

[29] Yu Z, Yang C, Bai X, Yao G, Qian X, Gao W, Huang Y, Tian X, Cheng S, Zheng Y. Risk factors for cholesterol polyp formation in the gallbladder are closely related to lipid metabolism. Lipids Health Dis. 2021;20(1):26. 33752687 10.1186/s12944-021-01452-6 PMC7983281

[30] Deng Z, Xuan Y, Li X, Crawford WJ, Yuan Z, Chen Z, Brooks A, Song Y, Wang H, Liang X, Chen T. Effect of metabolic syndrome components on the risk of malignancy in patients with gallbladder lesions. J Cancer. 2021;12(5):1531–7. 33531998 10.7150/jca.54617 PMC7847661

[31] Mellnick VM, Menias CO, Sandrasegaran K, Hara AK, Kielar AZ, Brunt EM, Doyle MB, Dahiya N, Elsayes KM. Polypoid lesions of the gallbladder: disease spectrum with pathologic correlation. Radiographics. 2015;35(2):387–99. 25763724 10.1148/rg.352140095

[32] Han S, Liu Y, Li X, Jiang X, Li B, Zhang C, Zhang J. Development and validation of a preoperative nomogram for predicting benign and malignant gallbladder polypoid lesions. Front Oncol. 2022;12: 800449. 35402267 10.3389/fonc.2022.800449 PMC8990775

[33] Li Q, Dou M, Zhang J, Jia P, Wang X, Lei D, Li J, Yang W, Yang R, Yang C, Zhang X, Hao Q, Geng X, Zhang Y, Liu Y, Guo Z, Yao C, Cai Z, Si S, Geng Z, Zhang D. A Bayesian network model to predict neoplastic risk for patients with gallbladder polyps larger than 10 mm based on preoperative ultrasound features. Surg Endosc. 2023;37(7):5453–63. 37041283 10.1007/s00464-023-10056-3

[34] Zhang X, Wang J, Wu B, Li T, Jin L, Wu Y, Gao P, Zhang Z, Qin X, Zhu C. A nomogram-based model and ultrasonic radiomic features for gallbladder polyp classification. J Gastroenterol Hepatol. 2022;37(7):1380–8. 35357026 10.1111/jgh.15841

[35] Wang X, Zhu JA, Liu YJ, Liu YQ, Che DD, Niu SH, Gao S, Chen DB. Conventional ultrasound combined with contrast-enhanced ultrasound in differential diagnosis of gallbladder cholesterol and adenomatous polyps (1–2 cm). J Ultrasound Med. 2022;41(3):617–26. 33938029 10.1002/jum.15740

[36] Stieger SM, Caskey CF, Adamson RH, Qin S, Curry FR, Wisner ER, Ferrara KW. Enhancement of vascular permeability with low-frequency contrast-enhanced ultrasound in the chorioallantoic membrane model. Radiology. 2007;243(1):112–21. 17392250 10.1148/radiol.2431060167

[37] Fei X, Cheng Z, Zhu L et al. A practical contrast-enhanced ultrasound risk prediction of gallbladder polyp: differentiation of adenoma from cholesterol polyp lesion. Abdom Radiol (NY) 2024. 10.1007/s00261-024-04566-4 39254706

[38] Kim T, Choi YH, Choi JH, et al. Gallbladder polyp classification in ultrasound images using an ensemble convolutional neural network model. J Clin Med. 2021;10:3585. 34441881 10.3390/jcm10163585 PMC8396835

