# Supplementary material for: Establishing a radiomics model using contrast-enhanced ultrasound for preoperative prediction of neoplastic gallbladder polyps exceeding 10 mm
Source: Eur J Med Res. 2025 Feb 3;30:66. doi: 10.1186/s40001-025-02292-1 (PMC11789348; doi:10.1186/s40001-025-02292-1)
Supplement: Supplementary file 1 — Additional file 1 [file 40001_2025_2292_MOESM1_ESM.docx]

**Supplemental Table1: Univariate and multivariate analyses of Clinic Signature**

| Feature name | **Univariable Analysis** | | **Multivariable Analysis** | |
| --- | --- | --- | --- | --- |
|  | OR | ***p***  **value** | OR | ***p***  **value** |
| HDL | 0.620(0.484,0.794) | 0.002 | 0.701(0.528,0.931) | 0.04 |
| smoking | 0.781(0.621,0.983) | 0.078 |  |  |
| cholecystitis | 0.789(0.602,1.035) | 0.149 |  |  |
| tumorhistory | 0.792(0.592,1.059) | 0.185 |  |  |
| hyperlipemia | 0.794(0.578,1.091) | 0.230 |  |  |
| drinking | 0.796(0.559,1.133) | 0.286 |  |  |
| Hightension | 0.865(0.692,1.081) | 0.282 |  |  |
| number | 0.889(0.762,1.036) | 0.203 |  |  |
| Cholesterol | 0.936(0.846,1.035) | 0.276 |  |  |
| LDL | 0.939(0.849,1.038) | 0.298 |  |  |
| Echo | 0.954(0.841,1.082) | 0.540 |  |  |
| Sex | 0.961(0.824,1.121) | 0.669 |  |  |
| gallbladder_wall | 0.981(0.776,1.240) | 0.892 |  |  |
| Base | 0.986(0.829,1.171) | 0.892 |  |  |
| CEA | 0.986(0.920,1.057) | 0.743 |  |  |
| Weight | 0.996(0.988,1.004) | 0.408 |  |  |
| Total bilirubin | 0.996(0.981,1.010) | 0.605 |  |  |
| CA199 | 1.000(0.998,1.001) | 0.575 |  |  |
| Neutrophil | 1.000(0.948,1.053) | 0.991 |  |  |
| Age | 1.001(0.994,1.008) | 0.787 |  |  |
| AST | 1.001(0.991,1.010) | 0.907 |  |  |
| Heigth | 1.003(0.993,1.013) | 0.645 |  |  |
| ALT | 1.003(0.998,1.007) | 0.350 |  |  |
| DM | 1.006(0.787,1.285) | 0.968 |  |  |
| Major_axis | 1.021(1.011,1.031) | 0.001 | 1.002(0.981,1.023) | 0.872 |
| The base width of CEUS | 1.027(1.014,1.041) | 0.001 | 1.01(0.989,1.03) | 0.439 |
| Broad diameter | 1.033(1.019,1.047) | 0.000 | 1.016(0.982,1.052) | 0.437 |
| glucose | 1.036(0.955,1.125) | 0.472 |  |  |
| The base width | 1.037(1.011,1.063) | 0.021 | 1.016(0.989,1.043) | 0.327 |
| Target lesion location | 1.060(0.974,1.154) | 0.250 |  |  |
| CDFI | 1.085(0.974,1.208) | 0.211 |  |  |
| Enhancement_level | 1.099(0.970,1.244) | 0.210 |  |  |
| Lymphocyte | 1.103(0.931,1.307) | 0.338 |  |  |
| Fatty liver | 1.111(0.940,1.313) | 0.297 |  |  |
| Triglyceride | 1.142(1.038,1.259) | 0.024 | 1.014(0.908,1.131) | 0.837 |
| Vascular_level | 1.157(1.021,1.311) | 0.055 |  |  |
| Enhancement_mode | 1.159(0.984,1.365) | 0.138 |  |  |
| Surface | 1.183(0.987,1.418) | 0.127 |  |  |
| Calculus | 1.231(0.977,1.551) | 0.137 |  |  |
| Shape | 1.241(1.009,1.525) | 0.086 |  |  |
| Close to gallbladder wall | 1.357(1.016,1.811) | 0.082 |  |  |

**Note**—NGB = neoplastic GBP, ALT = alanine aminotransferase, AST = aspartate aminotransferase, CEUS= contrast-enhanced ultrasound, CDFI = color doppler flow imaging, *P* value for comparisons between non-NGB and NGB patients.

**Supplemental Table2 :** Performance of clinical signature in the training cohort

| **model_name** | **Accuracy** | **AUC** | **95% CI** | **Sensitivity** | **Specificity** | **PPV** | **NPV** | **Precision** | **Recall** | **F1** | **Threshold** |
| --- | --- | --- | --- | --- | --- | --- | --- | --- | --- | --- | --- |
| LR-training | 0.723 | 0.787 | 0.6677 - 0.9066 | 0.778 | 0.719 | 0.424 | 0.920 | 0.424 | 0.778 | 0.549 | 0.200 |
| LR-test | 0.806 | 0.786 | 0.5807 - 0.9907 | 0.714 | 0.828 | 0.500 | 0.923 | 0.500 | 0.714 | 0.588 | 0.202 |
| SVM-training | 0.807 | 0.772 | 0.6286 - 0.9150 | 0.722 | 0.831 | 0.542 | 0.915 | 0.542 | 0.722 | 0.619 | 0.193 |
| SVM-test | 0.861 | 0.717 | 0.4703 - 0.9632 | 0.571 | 0.931 | 0.667 | 0.900 | 0.667 | 0.571 | 0.615 | 0.196 |
| KNN-train | 0.831 | 0.855 | 0.7695 - 0.9408 | 0.722 | 0.862 | 0.591 | 0.918 | 0.591 | 0.722 | 0.650 | 0.400 |
| KNN-test | 0.778 | 0.734 | 0.5606 - 0.9073 | 0.429 | 0.862 | 0.429 | 0.862 | 0.429 | 0.429 | 0.429 | 0.400 |
| RandomForest-training | 0.964 | 0.981 | 0.9526 - 1.0000 | 0.944 | 0.969 | 0.895 | 0.984 | 0.895 | 0.944 | 0.919 | 0.400 |
| RandomForest-test | 0.722 | 0.704 | 0.4875 - 0.9213 | 0.714 | 0.750 | 0.385 | 0.913 | 0.385 | 0.714 | 0.500 | 0.184 |
| ExtraTrees-training | 0.988 | 0.997 | 0.9895 - 1.0000 | 0.944 | 1.000 | 1.000 | 0.985 | 1.000 | 0.944 | 0.971 | 1.000 |
| ExtraTrees-test | 0.750 | 0.655 | 0.4311 - 0.8792 | 0.571 | 0.852 | 0.400 | 0.885 | 0.400 | 0.571 | 0.471 | 0.122 |
| XGBoost-training | 0.892 | 0.917 | 0.8522 - 0.9820 | 0.722 | 0.938 | 0.765 | 0.924 | 0.765 | 0.722 | 0.743 | 0.348 |
| XGBoost-test | 0.778 | 0.732 | 0.5494 - 0.9137 | 0.571 | 0.889 | 0.444 | 0.889 | 0.444 | 0.571 | 0.500 | 0.189 |
| LightGBM-training | 0.783 | 0.783 | 0.6728 - 0.8939 | 0.556 | 0.902 | 0.500 | 0.873 | 0.500 | 0.556 | 0.526 | 0.316 |
| LightGBM-test | 0.833 | 0.754 | 0.5484 - 0.9590 | 0.571 | 1.000 | 0.571 | 0.897 | 0.571 | 0.571 | 0.571 | 0.360 |
| MLP-training | 0.747 | 0.756 | 0.6286 - 0.8842 | 0.722 | 0.766 | 0.448 | 0.907 | 0.448 | 0.722 | 0.553 | 0.233 |
| MLP-test | 0.833 | 0.766 | 0.5446 - 0.9874 | 0.714 | 0.862 | 0.556 | 0.926 | 0.556 | 0.714 | 0.625 | 0.233 |

Note-SVM=Support Vector Machine, LR=Logistic Regression, MLP=Multilayer Perceptron, KNN=k-Nearest Neighbors, XGBoost=eXtreme Gradient Boosting, AUC = area under the receiver operating characteristic curve, CI=confidence interval.

**Supplemental Table3 :** Performance of conventional ultrasonic radiomic signature in the training cohort

| **model_name** | **Accuracy** | **AUC** | **95% CI** | **Sensitivity** | **Specificity** | **PPV** | **NPV** | **Precision** | **Recall** | **F1** | **Threshold** |
| --- | --- | --- | --- | --- | --- | --- | --- | --- | --- | --- | --- |
| LR-training | 0.819 | 0.848 | 0.7548 - 0.9409 | 0.722 | 0.846 | 0.565 | 0.917 | 0.565 | 0.722 | 0.634 | 0.295 |
| LR-test | 0.750 | 0.734 | 0.5552 - 0.9128 | 0.714 | 0.786 | 0.417 | 0.917 | 0.417 | 0.714 | 0.526 | 0.231 |
| SVM-training | 0.940 | 0.950 | 0.8704 - 1.0000 | 0.944 | 0.938 | 0.810 | 0.984 | 0.810 | 0.944 | 0.872 | 0.197 |
| SVM-test | 0.500 | 0.645 | 0.4275 - 0.8631 | 1.000 | 0.379 | 0.280 | 1.000 | 0.280 | 1.000 | 0.437 | 0.174 |
| KNN-training | 0.819 | 0.877 | 0.8067 - 0.9471 | 0.833 | 0.828 | 0.556 | 0.946 | 0.556 | 0.833 | 0.667 | 0.400 |
| KNN-test | 0.444 | 0.515 | 0.2943 - 0.7353 | 0.714 | 0.407 | 0.217 | 0.846 | 0.217 | 0.714 | 0.333 | 0.200 |
| RandomForest-training | 1.000 | 1.000 | 1.0000 - 1.0000 | 1.000 | 1.000 | 1.000 | 1.000 | 1.000 | 1.000 | 1.000 | 0.500 |
| RandomForest-test | 0.556 | 0.640 | 0.4218 - 0.8590 | 0.857 | 0.483 | 0.286 | 0.933 | 0.286 | 0.857 | 0.429 | 0.200 |
| ExtraTrees-training | 1.000 | 1.000 | 1.0000 - 1.0000 | 1.000 | 1.000 | 1.000 | 1.000 | 1.000 | 1.000 | 1.000 | 1.000 |
| ExtraTrees-test | 0.778 | 0.603 | 0.3390 - 0.8679 | 0.429 | 0.893 | 0.429 | 0.862 | 0.429 | 0.429 | 0.429 | 0.400 |
| XGBoost-training | 1.000 | 1.000 | 1.0000 - 1.0000 | 1.000 | 1.000 | 1.000 | 1.000 | 1.000 | 1.000 | 1.000 | 0.585 |
| XGBoost-test | 0.778 | 0.576 | 0.2846 - 0.8681 | 0.429 | 0.862 | 0.429 | 0.862 | 0.429 | 0.429 | 0.429 | 0.299 |
| LightGBM-training | 0.831 | 0.925 | 0.8702 - 0.9803 | 0.944 | 0.800 | 0.567 | 0.981 | 0.567 | 0.944 | 0.708 | 0.293 |
| LightGBM-test | 0.694 | 0.589 | 0.2708 - 0.9065 | 0.571 | 0.724 | 0.333 | 0.875 | 0.333 | 0.571 | 0.421 | 0.293 |
| MLP-training | 0.759 | 0.855 | 0.7723 - 0.9371 | 0.944 | 0.708 | 0.472 | 0.979 | 0.472 | 0.944 | 0.630 | 0.253 |
| MLP-test | 0.583 | 0.709 | 0.4843 - 0.9344 | 0.857 | 0.536 | 0.300 | 0.937 | 0.300 | 0.857 | 0.444 | 0.229 |

Note-SVM=Support Vector Machine, LR=Logistic Regression, MLP=Multilayer Perceptron, KNN=k-Nearest Neighbors, XGBoost=eXtreme Gradient Boosting, AUC = area under the receiver operating characteristic curve, CI=confidence interval.

**Supplemental Table4 :** Performance of contrast-enhanced ultrasonic radiomic signature in the training cohort

| **model_name** | **Accuracy** | **AUC** | **95% CI** | **Sensitivity** | **Specificity** | **PPV** | **NPV** | **Precision** | **Recall** | **F1** | **Threshold** |
| --- | --- | --- | --- | --- | --- | --- | --- | --- | --- | --- | --- |
| LR-training | 0.819 | 0.892 | 0.8152 - 0.9694 | 0.889 | 0.800 | 0.552 | 0.963 | 0.552 | 0.889 | 0.681 | 0.203 |
| LR-test | 0.722 | 0.798 | 0.6418 - 0.9543 | 1.000 | 0.655 | 0.412 | 1.000 | 0.412 | 1.000 | 0.583 | 0.152 |
| SVM-training | 0.952 | 0.912 | 0.7968 - 1.0000 | 0.889 | 0.969 | 0.889 | 0.969 | 0.889 | 0.889 | 0.889 | 0.195 |
| SVM-test | 0.611 | 0.788 | 0.6119 - 0.9645 | 1.000 | 0.517 | 0.333 | 1.000 | 0.333 | 1.000 | 0.500 | 0.164 |
| KNN-training | 0.554 | 0.805 | 0.7124 - 0.8970 | 1.000 | 0.431 | 0.327 | 1.000 | 0.327 | 1.000 | 0.493 | 0.200 |
| KNN-test | 0.556 | 0.793 | 0.6298 - 0.9564 | 1.000 | 0.448 | 0.304 | 1.000 | 0.304 | 1.000 | 0.467 | 0.200 |
| RandomForest-training | 1.000 | 1.000 | 1.0000 - 1.0000 | 1.000 | 1.000 | 1.000 | 1.000 | 1.000 | 1.000 | 1.000 | 0.500 |
| RandomForest-test | 0.611 | 0.818 | 0.6572 - 0.9783 | 1.000 | 0.517 | 0.333 | 1.000 | 0.333 | 1.000 | 0.500 | 0.200 |
| ExtraTrees-trainng | 1.000 | 1.000 | 1.0000 - 1.0000 | 1.000 | 1.000 | 1.000 | 1.000 | 1.000 | 1.000 | 1.000 | 1.000 |
| ExtraTrees-test | 0.861 | 0.667 | 0.4483 - 0.8867 | 0.286 | 1.000 | 1.000 | 0.853 | 1.000 | 0.286 | 0.444 | 0.800 |
| XGBoost-training | 1.000 | 1.000 | 1.0000 - 1.0000 | 1.000 | 1.000 | 1.000 | 1.000 | 1.000 | 1.000 | 1.000 | 0.452 |
| XGBoost-test | 0.833 | 0.828 | 0.6595 - 0.9957 | 0.714 | 0.862 | 0.556 | 0.926 | 0.556 | 0.714 | 0.625 | 0.308 |
| LightGBM-training | 0.867 | 0.924 | 0.8677 - 0.9811 | 0.944 | 0.873 | 0.630 | 0.982 | 0.630 | 0.944 | 0.756 | 0.261 |
| LightGBM-test | 0.694 | 0.717 | 0.4865 - 0.9470 | 0.857 | 0.704 | 0.375 | 0.950 | 0.375 | 0.857 | 0.522 | 0.252 |
| MLP-training | 0.855 | 0.900 | 0.8162 - 0.9838 | 0.833 | 0.862 | 0.625 | 0.949 | 0.625 | 0.833 | 0.714 | 0.276 |
| MLP-test | 0.556 | 0.744 | 0.5576 - 0.9301 | 1.000 | 0.448 | 0.304 | 1.000 | 0.304 | 1.000 | 0.467 | 0.182 |

Note-SVM=Support Vector Machine, LR=Logistic Regression, MLP=Multilayer Perceptron, KNN=k-Nearest Neighbors, XGBoost=eXtreme Gradient Boosting, AUC = area under the receiver operating characteristic curve, CI=confidence interval.

**Supplemental Table5 :** Performance of conventional ultrasonic and contrast-enhanced ultrasonic radiomic signature in the training cohort

| **model_name** | **Accuracy** | **AUC** | **95% CI** | **Sensitivity** | **Specificity** | **PPV** | **NPV** | **Precision** | **Recall** | **F1** | **Threshold** |
| --- | --- | --- | --- | --- | --- | --- | --- | --- | --- | --- | --- |
| LR-training | 0.867 | 0.922 | 0.8598 - 0.9846 | 0.889 | 0.862 | 0.640 | 0.966 | 0.640 | 0.889 | 0.744 | 0.188 |
| LR-test | 0.806 | 0.808 | 0.6288 - 0.9869 | 0.857 | 0.793 | 0.500 | 0.958 | 0.500 | 0.857 | 0.632 | 0.233 |
| SVM-training | 0.976 | 0.963 | 0.8990 - 1.0000 | 0.889 | 1.000 | 1.000 | 0.970 | 1.000 | 0.889 | 0.941 | 0.364 |
| SVM-test | 0.833 | 0.714 | 0.4719 - 0.9567 | 0.571 | 0.897 | 0.571 | 0.897 | 0.571 | 0.571 | 0.571 | 0.423 |
| KNN-training | 0.735 | 0.915 | 0.8599 - 0.9708 | 1.000 | 0.662 | 0.450 | 1.000 | 0.450 | 1.000 | 0.621 | 0.200 |
| KNN-test | 0.667 | 0.734 | 0.5562 - 0.9117 | 0.857 | 0.643 | 0.353 | 0.947 | 0.353 | 0.857 | 0.500 | 0.200 |
| RandomForest-training | 1.000 | 1.000 | 1.0000 - 1.0000 | 1.000 | 1.000 | 1.000 | 1.000 | 1.000 | 1.000 | 1.000 | 0.500 |
| RandomForest-test | 0.722 | 0.704 | 0.4770 - 0.9318 | 0.571 | 0.786 | 0.364 | 0.880 | 0.364 | 0.571 | 0.444 | 0.300 |
| ExtraTrees-training | 1.000 | 1.000 | 1.0000 - 1.0000 | 1.000 | 1.000 | 1.000 | 1.000 | 1.000 | 1.000 | 1.000 | 1.000 |
| ExtraTrees-test | 0.833 | 0.828 | 0.6459 - 1.0000 | 0.571 | 0.897 | 0.571 | 0.897 | 0.571 | 0.571 | 0.571 | 0.400 |
| XGBoost-training | 1.000 | 1.000 | 1.0000 - 1.0000 | 1.000 | 1.000 | 1.000 | 1.000 | 1.000 | 1.000 | 1.000 | 0.541 |
| XGBoost-test | 0.528 | 0.675 | 0.4393 - 0.9105 | 0.857 | 0.448 | 0.273 | 0.929 | 0.273 | 0.857 | 0.414 | 0.115 |
| LightGBM-training | 0.880 | 0.953 | 0.9115 - 0.9936 | 1.000 | 0.846 | 0.643 | 1.000 | 0.643 | 1.000 | 0.783 | 0.237 |
| LightGBM-test | 0.472 | 0.687 | 0.4678 - 0.9065 | 1.000 | 0.345 | 0.269 | 1.000 | 0.269 | 1.000 | 0.424 | 0.123 |
| MLP-training | 0.867 | 0.920 | 0.8544 - 0.9849 | 0.833 | 0.877 | 0.652 | 0.950 | 0.652 | 0.833 | 0.732 | 0.273 |
| MLP-test | 0.833 | 0.823 | 0.6348 - 1.0000 | 0.714 | 0.862 | 0.556 | 0.926 | 0.556 | 0.714 | 0.625 | 0.267 |

Note-SVM=Support Vector Machine, LR=Logistic Regression, MLP=Multilayer Perceptron, KNN=k-Nearest Neighbors, XGBoost=eXtreme Gradient Boosting, AUC = area under the receiver operating characteristic curve, CI=confidence interval.
